# Dry-Transferred MoS_2_ Films on PET with Plasma Patterning for Full-Bridge Strain-Gauge Sensors

**DOI:** 10.3390/s26020585

**Published:** 2026-01-15

**Authors:** Jinkyeong Kim, Minjae Lee, Wooseung Lee, Minseok Lee, Chang-Mo Kang, Daewoong Jung, Hyunwoo Son, Eunyoung Kim, Sangwoo Chae, Joonhyub Kim

**Affiliations:** 1 The Department of Nanomechatronics Engineering, Pusan National University, Busandaehak-ro 63 beon-gil 2, Geumjeong-gu, Busan 46241, Republic of Korea; wlsrud02@pusan.ac.kr (J.K.); ericmin3411@pusan.ac.kr (M.L.); yoshino22@pusan.ac.kr (W.L.); ehfrhfo0203@pusan.ac.kr (M.L.); dwjung@pusan.ac.kr (D.J.); 2 The Department of Electronic Engineering, Gyeongsang National University, Jinju 52828, Republic of Korea; sonhyunwoo@gnu.ac.kr; 3 The Department of Physical Therapy, Masan University, Changwon 51217, Republic of Korea; 4 Institute of Innovation for Future Society, Nagoya University, Furo-cho, Chikusa-ku, Nagoya 464-8603, Japan

**Keywords:** MoS_2_, sensor, strain gauge, plasma patterning

## Abstract

In this study, a high-performance MoS_2_-based strain-gauge pressure was sensor fabricated entirely below 80 °C, enabling direct integration onto flexible polyethylene terephthalate (PET) substrates. The sensor comprised a three-layer MoS_2_ channel (~2 nm) patterned via dry transfer and O_2_/Ar plasma etching, interfaced with Cr/Au electrodes. This wafer-scale and cost-effective fabrication route preserves the crystallinity of the film and prevents substrate degradation. The sensor achieved a gauge factor of ~104 under compression, representing a fifty-fold improvement over conventional metal foil gauges (~2), with a linear response across both compressive and tensile regimes. Mechanical robustness was confirmed through repeated bending and tape adhesion tests, with no degradation in electrical performance. When configured as a Wheatstone bridge, this device exhibits normalized sensitivity suitable for real-time monitoring, with response and recovery times below 200 ms. These results establish O_2_/Ar-plasma-patterned MoS_2_ architectures as a scalable, cost-effective platform for next-generation flexible sensors, outperforming metal-foil technology in applications including seat-occupancy detection, wearable physiological monitoring, and tactile interfaces for soft robotics.

## 1. Introduction

The expansion of wearable electronics and human–machine interfaces demands next-generation sensors that are lightweight and flexible, with high reliability [1,2]. In healthcare, skin-conformal flexible sensors must continuously monitor physiological signals such as pulse, respiration, and joint motion in real time. In robotics, artificial tactile elements must accurately feedback external pressure and touch information [3,4]. In the automotive industry, low-power, high-sensitivity pressure sensors are essential for seat occupancy detection and airbag deployment optimization [5]. Because these applications generally involve attaching sensors to curved or deformable surfaces, the devices must respond precisely to small mechanical deformations while preserving electrical and mechanical stability under repeated bending and twisting [6,7].

Conventional metal foil strain gauges have served as the industrial standard for decades and offer excellent linearity. However, their gauge factor (GF) of approximately 2 limits the detection of small strains, and repeated deformation often induces rapid electrical fatigue and mechanical degradation [8]. To overcome these limitations, carbon nanotube (CNT) and graphene-based sensors, which exhibit good flexibility and high electrical conductivity, have been explored [9]. However, network nonuniformity and noise can compromise linearity. Doped polymer sensors offer high processability but face challenges in terms of long-term stability [10]. These limitations highlight the need for channel materials that simultaneously deliver fast responses, linearity, and stability.

Consequently, two-dimensional transition-metal dichalcogenides (TMDs) have emerged as promising candidates for next-generation strain sensors [11,12]. Molybdenum disulfide (MoS_2_) combines an atomically thin thickness and high tensile strength with a piezoresistive effect that depends on the crystallographic orientation of the band structure. Tuning the number of layers can increase the GF to hundreds [13,14,15]. Monolayers offer high mobility and optical transparency, but reduce mechanical robustness, whereas bulk layers provide mechanical stability at the expense of sensitivity [16,17,18]. Three-layer MoS_2_ exhibits improved mobility relative to monolayers, along with optimized on-current density and charge injection characteristics, making it attractive for high-sensitivity strain gauges. However, many previously reported high-GF MoS_2_ sensors rely on rigid substrates or high-temperature processing (<150 °C), which makes them less compatible with low-temperature flexible substrates [19]. Other TMDs such as WS_2_ and WSe_2_ have also been investigated, but MoS_2_ remains the most promising in terms of synthetic accessibility and stability.

Among the flexible substrates, polyethylene terephthalate (PET) is attractive for large-area commercialization owing to its low cost, chemical resistance, and high optical transmittance. However, the glass transition temperature of PET (Tg ≈ 80 °C) is lower than the typical temperatures used for metal deposition and photolithography, rendering PET susceptible to thermal deformation and mechanical cracking [20]. In addition, residual solvents and polymeric residues from solution-based transfer are a major source of signal noise, as they can increase surface roughness and interfacial contact resistance. Dry transfer strategies have recently been proposed to mitigate these issues; however, applications of PET remain limited [21].

We integrated three-layer (~2 nm) chemical vapor deposition (CVD)-grown MoS_2_ onto PET using a dry transfer method, after defining Cr/Au electrodes via electron beam evaporation and subsequently applying plasma etching to form gauge patterns. This approach follows a low-temperature, low-damage process flow that is compatible with flexible substrates. Furthermore, we constructed a full-Wheatstone bridge on a PET diaphragm and systematically quantified its response and recovery times, cycling reliability, and sensitivity to kilopascal-range pressure variations.

A gauge factor of approximately 104 was achieved under compressive strain with response and recovery times below 200 ms while maintaining electrical and mechanical stability after repeated bending and adhesion tests. These findings demonstrate that an integrated approach combining low-temperature processing, a 2D channel, and a bridge configuration enables sensitivity improvements of more than an order of magnitude over metal foil gauges on flexible substrates. Design guidelines for high-sensitivity, low-power, and real-time pressure monitoring are provided in practical environments such as vehicle seat occupancy detection, wearable healthcare, and tactile sensing for soft robotics.

## 2. Experimental Methods

### 2.1. Materials and Characterization

Three-layer MoS_2_ synthesized by CVD was purchased from 6Carbon Co. (Shenzhen, China). A PET film with a thickness of 125 μm was obtained from Graphene Platform (Tokyo, Japan) as the flexible substrate. Metal electrodes were deposited using an electron-beam evaporation system (KVET, Korea Vacuum Tech, Gimpo, Republic of Korea). All the chemical solutions used in the cleaning process were purchased from Sigma-Aldrich (St. Louis, MO, USA; ACS-grade purity).

Sample thickness and surface morphology were characterized by atomic force microscopy (AFM; Shimadzu, Kyoto, Japan). Measurements were performed at room temperature (25 °C) with a resonance frequency of 320 kHz and scan rate of 0.5 Hz. The scan area was set to 10 × 10 µm^2^.

For pressure application, the samples were fabricated in a rectangular shape with dimensions of 2 cm (width) × 7 cm (length). The mechanical strain of the samples was characterized using a universal testing machine (MTS landmark 100 kN; MTS Systems Corporation, Eden Prairie, MN, USA), to determine the Young’s modulus (E = stress/strain). The electrical properties under an applied strain were evaluated using an LCR meter (Hioki, Nagano, Japan).

The plasma reactor was operated in capacitively coupled plasma (CCP) mode using parallel-plate reactive ion etching (RIE) electrodes driven at a radio frequency (RF) of 13.56 MHz. During processing, the forward RF power was set to 30 W. An aluminum chamber with internal dimensions of 200 mm (width) × 220 mm (depth) × 160 mm (height) was evacuated to a base pressure of 0.05 Torr, and process pressure was controlled using an automatic pressure controller (APC). A mixed gas of oxygen and argon was introduced into the chamber at a ratio of O_2_/Ar = 2:1 with a total flow rate of 100 standard cubic centimeters per minute (sccm).

The characteristics of MoS_2_ according to layer number were analyzed by X-ray photoelectron spectroscopy (XPS, Thermo Fisher Scientific, Waltham, MA, USA). The binding energies were calibrated with reference to the C 1s peak at 284.6 eV. The structural characteristics of MoS_2_ after the dry transfer process were further characterized by Raman spectroscopy (LabRAM; Horiba, Kyoto, Japan), using a 532 nm laser excitation source.

### 2.2. Strain Sensor Fabrication

**Figure 1:**
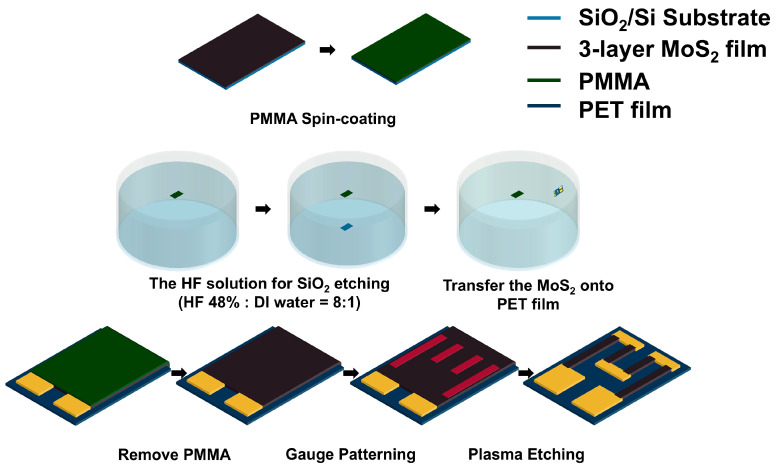
Low-temperature fabrication schematic of a trilayer MoS_2_ strain gauge on PET: Cr/Au electrodes, PMMA-assisted dry transfer, and O_2_/Ar plasma patterning.

To fabricate the strain gauge, a three-layer MoS_2_ (approximately 2 nm) thin film grown by CVD on a SiO_2_/Si substrate was dry transferred onto a flexible PET film. The PET film (5 × 5 mm^2^) was first attached to a dummy Si wafer using Kapton tape to provide mechanical support during the subsequent deposition steps. Metal electrodes (Cr/Au, 10 nm/200 nm) were then deposited via electron-beam evaporation. The electrode stack was patterned using gold (AT-409, Jeonyoung Co., Ltd., Gunsan, Republic of Korea) and chromium etchants (Sigma-Aldrich, St. Louis, MO, USA; ACS-grade purity) diluted 1:10 (*v*/*v*) with deionized water, with etching times of 7 min (Au) and 30 s (Cr). The fabricated Au electrodes were further cleaned in piranha solution (H_2_SO_4_:H_2_O_2_ = 3:1 *v*/*v*) for 15 min to remove organic residues. The strain gauge was designed with a length of 2.4 mm and a width of 0.4 mm, resulting in an overall sensor size of 3 mm × 4 mm. The step-by-step fabrication procedure for this device is detailed in Figure 1.

For MoS_2_ transfer, a poly(methyl methacrylate) (PMMA) support layer was spin-coated onto the CVD-grown three-layer MoS_2_/SiO_2_/Si substrate at 3000 rpm for 60 s. The sample was cut using a diamond scriber and immersed in a buffered HF solution (48 wt.% HF, diluted 1:10 *v*/*v* with deionized water) to etch the SiO_2_ layer and release the MoS_2_/PMMA stack. The floating stack was then transferred to a PET substrate and dried.

After the transfer, the negative photoresist was spin-coated at 4000 rpm for 40 s. The gauge area was then patterned by O_2_/Ar plasma etching to remove unwanted MoS_2_ outside the channel. The final three-layer MoS_2_ strain gauge maintained structural integrity and Raman spectral characteristics, confirming that the transfer and patterning processes did not degrade the intrinsic properties of the MoS_2_ film.

## 3. Results and Discussion

**Figure 2:**
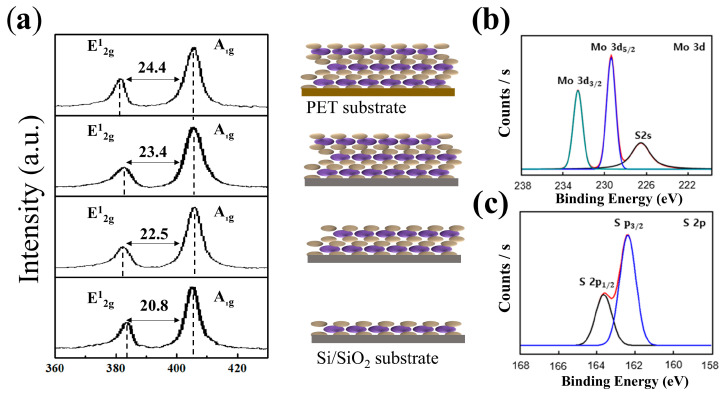
(**a**) Experimentally measured Raman spectra for varying MoS_2_ layer numbers. (**b**,**c**) Mo^4+^ and S^2−^ states characteristic of MoS_2_ by XPS after transfer and plasma patterning.

Previous studies have shown that MoS_2_ exhibits in-plane (E^1^_2g_) and out-of-plane (A_1g_) vibrational mode characteristics in the Raman shift range of 370–420 cm^−1^ [22]. Figure 2a compares the Raman spectra of CVD-grown monolayer, bilayer, and trilayer MoS_2_ on SiO_2_/Si substrates with those of trilayer specimens transferred onto PET and patterned into gauge structures. With increasing layer number, the A_1g_ mode shifts to higher wavenumbers, whereas the E_2_g mode shifts to lower wavenumbers, resulting in a peak separation increase Δω [22,23,24,25,26]. This trend arises because interlayer van der Waals coupling and long-range interactions strengthen the restoring force for out-of-plane vibrations, whereas in-plane vibrations weaken Coulombic screening.

For trilayer MoS_2_ on SiO_2_/Si, ωE ≈ 382.9 cm^−1^ and ωA ≈ 401.6 cm^−1^, yielding Δω ≡ ωA − ωE ≈ 23.2 cm^−1^. After transfer to PET and patterning, changes in ωE and ωA remained within measurement uncertainty (±0.5 cm^−1^), and full width at half maximum (FWHM) values were maintained at 4.3 cm^−1^ (E^1^_2g_) and 5.0 cm^−1^ (A_1g_), respectively. Variations in the intensity ratio (I_A_/I_E_) and Δω were within 2% (Figure 2a), confirming that both layer number and crystallographic integrity were preserved. The observed layer-dependent increases in Δω (monolayer: 18.6 cm^−1^, bilayer: 22.7 cm^−1^, trilayer: 23.2 cm^−1^) are consistent with those of the literature.

This trilayer configuration offers distinct advantages in sensor applications. Recent reports indicate that room-temperature field-effect mobility increases from approximately 80 cm^2^ V^−1^ s^−1^ for monolayer to approximately 145 cm^2^ V^−1^ s^−1^ for trilayer MoS_2_, with corresponding on-current density improvements [27]. Li et al. demonstrated that few-layer MoS_2_ exhibits reduced contact barriers and enhanced surface trap screening, thereby facilitating charge injection and lowering low-frequency noise [28]. Although our device is based on a resistive gauge configuration rather than a field-effect transistor, the trilayer MoS_2_ structure still provides enhanced signal-to-noise ratio because of the higher gauge factor and reduced noise sources, enabling reliable operation even under low-bias voltage.

Plasma etching of MoS_2_ by O_2_/Ar proceeds through a stepwise mechanism in which sulfur (S) is preferentially removed, followed by residual molybdenum (Mo). Specifically, MoS_2_ decomposes into Mo and S through interactions with reactive species (O_2_^+^, O^+^, Ar^+^) in the plasma. As the resulting metallic Mo exhibited relatively low chemical reactivity, it was subsequently removed by physical sputtering. This process includes a physical removal mechanism. Therefore, MoS_2_ etching follows a two-stage pathway:

$${MoS}_{2\left(solid\right)}\overset{O_{2}^{+},O^{+},{Ar}^{+}}{\to }{MoS}_{\left(solid\right)}+S_{\left(physical\right)}$$

$${MoS}_{2\left(solid\right)}+2O\overset{O_{2}^{+},O^{+},{Ar}^{+}}{\to }{MoS}_{\left(solid\right)}+{SO}_{2\left(chemical\right)}$$

selective sulfur removal followed by sputtering-based Mo removal [29].

Figure 2b,c show high-resolution XPS profiles of the three-layer MoS_2_ strain gauge. In the Mo 3d core-level spectrum (Figure 2b), two distinct peaks are observed at 229.2 eV (Mo 3d_5_/_2_) and 232.3 eV (Mo 3d_3_/_2_), corresponding to the characteristic doublet of Mo^4+^ in MoS_2_. In the Mo 3d region, only the characteristic Mo^4+^ doublet of 2H-MoS_2_ is observed at 229–233 eV, while no peaks appear in the 235–236 eV range typically associated with Mo^6+^ (MoO_3_). The absence of Mo^6+^ signals indicates that the MoS_2_ surface remains minimally oxidized during processing. This interpretation is further supported by the sharp S 2p doublet at 161–163 eV, confirming the dominance of S^2−^ states. In the S 2p core-level spectrum, two peaks appear at 162.0 eV (S 2p_3/2_) and 163.3 eV (S 2p_1/2_), attributed to sulfide (S^2−^) species. The measured binding energies are in excellent agreement with reported values, confirming the chemical stability of the trilayer MoS_2_ after the transfer process [30,31].

**Figure 3:**
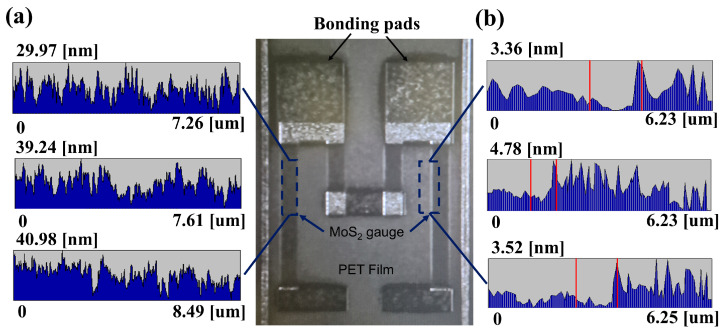
AFM characterization of the MoS_2_ strain gauge after O_2_/Ar plasma processing. (**a**) Surface roughness profiles measured exclusively on the MoS_2_ gauge region. (**b**) Height profiles acquired across the boundary between the MoS_2_ gauge and the PET substrate.

Figure 3a shows AFM analysis of the specimen surface after plasma patterning. The average arithmetic roughness (R_a_) measured was 0.38 ± 0.05 nm, and the root mean square roughness (Rq) was 0.27 ± 0.03 nm. These values are similar to or slightly lower than previously reported ranges (0.4–0.6 nm) for CVD-grown polycrystalline MoS_2_, suggesting that the dry transfer process effectively suppresses residual solvents and polymer byproducts. Linear profiles acquired across edge regions indicated an average step height of 2.49 ± 0.12 nm per layer. The AFM step height (~2.49 nm) of the trilayer MoS_2_ corresponds well to the typical range (2.4–3.0 nm) reported for transferred MoS_2_ on non-ideal substrates, where interfacial water, polymer residues, and substrate roughness lead to larger apparent thicknesses than the theoretical interlayer spacing [32]. The thickness variation (σ_thickness) across the entire scan area was less than 0.2 nm, corresponding to a standard deviation below 3.5%, demonstrating excellent thickness uniformity of the transferred three-layer MoS_2_ film.

**Figure 4:**
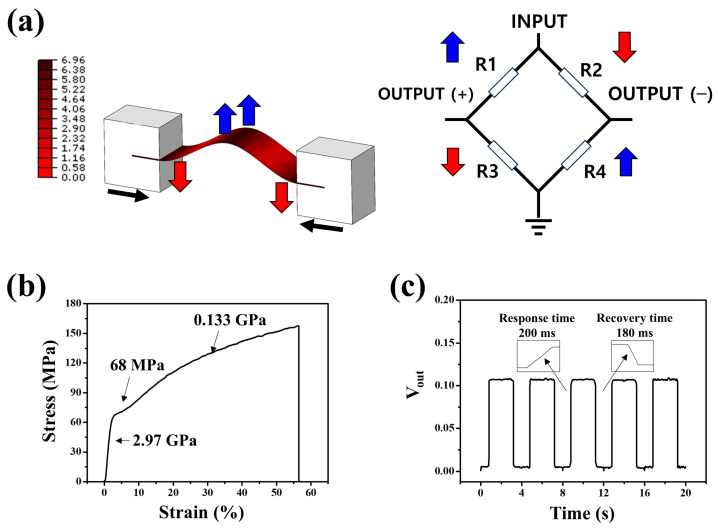
(**a**) Abaqus finite-element model of the PET film (2 × 7 mm^2^) under imposed displacement, simulated to identify suitable locations for strain sensor placement. (**b**) Stress–strain curve of the MoS_2_/PET sample. (**c**) Time-dependent electrical response of the sensor measured using the DC-motor-based actuation system.

A displacement-controlled bending jig was constructed using a DC motor-driven linear stage to validate the performance of the MoS-based strain gauge. When the left and right stages were moved by the motor while both ends of the PET film were fixed; tensile (positive) strain was generated at the film center, and compressive (negative) strain near the fixed ends. The maximum principal stress locations were estimated using finite element analysis (FEA software Abaqus 2020), and gauge attachment positions were determined based on the stress distribution results shown in Figure 4a. Four MoS_2_ gauges were arranged to form a full Wheatstone bridge configuration, with R_1_ and R_4_ attached in the tensile region and R_2_ and R_3_ in the compressive region. Consequently, gauges in the tensile region (R_1_, R_4_) exhibited increasing resistance with increasing strain, whereas gauges in the compressive region (R_2_, R_3_) exhibited decreasing resistance. Therefore, this layout ensures a symmetric bridge response that enhances sensitivity and compensates for thermal drift.

**Figure 5:**
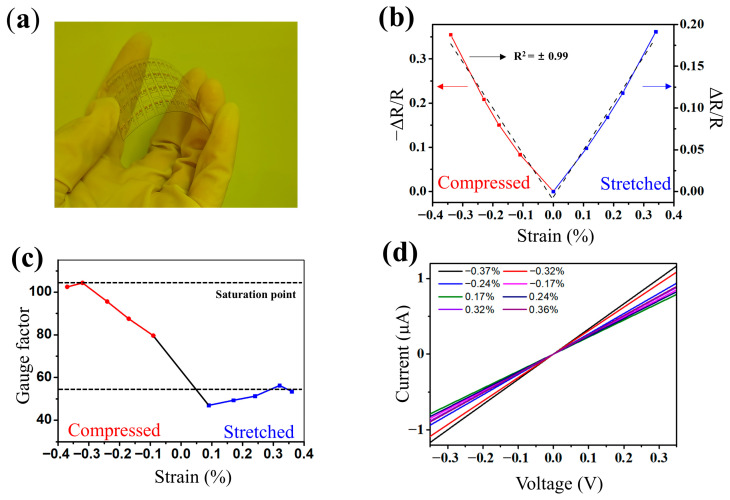
(**a**) Photograph of the MoS_2_ strain gauge on a PET substrate under bending. (**b**) Relative resistance change (ΔR/R) versus strain under compressive and tensile loading, showing highly linear behavior with a correlation coefficient of R^2^ = 0.99. (**c**) Gauge factor versus strain, showing high sensitivity and saturation beyond ~0.32% (GF ≈ 104). (**d**) I–V curves at selected applied strains.

The gauges were attached to the PET surface using a double-sided tape (467 MP, 3 M). A bridge excitation voltage of 5 V DC was applied and the output voltage was measured using a digital multimeter (DMM) after 1 h of stabilization at a set temperature. Figure 4b shows the stress–strain curve of the MoS_2_/PET sample measured using a universal testing machine (UTM). The result reflects the mechanical response of the PET substrate supporting the MoS_2_ strain gauge under uniaxial tensile loading. A linear elastic region is observed at low strain, followed by a nonlinear deformation regime at higher strain levels, indicating typical polymer-dominated mechanical behavior. This measurement provides a mechanical basis for correlating the applied strain with the electrical response of the MoS_2_-based strain sensor. Figure 5c shows the dynamic response test results obtained using a linear actuator. The sensor exhibited a stable response to repetitive deformation with response and recovery times of ~200 and ~180 ms, respectively. The response time was defined as the time required for the output voltage to reach 90% of its steady-state value after the application of deformation, while the recovery time was defined as the time required for the signal to return to 10% of its maximum value after load removal. Quantitatively, the response time *t_res_* was defined as the time at which the output voltage satisfies

(1) $$V\left(t_{res}\right)=0.9\;V_{max},$$

where *V_max_* denotes the steady-state output voltage.

Figure 5a shows an optical image of the fabricated MoS-based strain gauge under bending. Figure 5b shows the resistance change ratio (ΔR/R) under compressive and tensile strains. The resistance decreases in the compressive region and increases in the tensile region, exhibiting symmetric behavior in both directions. In addition, a strong linear correlation between ΔR/R and applied strain is observed in both regimes, with a correlation coefficient of $R^{2}=0.99$, indicating highly stable and reproducible strain-dependent electrical responses. Figure 5c shows the variation in gauge factor measured in the strain range from approximately −0.4% to +0.4%. The gauge factor was calculated using the following equation:

(2) $$GF=\frac{∆R/R}{\epsilon }$$

where ΔR/R is the resistance change ratio, and ε is the applied strain. For the fabricated sensor, deviations from linearity became apparent beyond 0.32% strain, indicating saturation, and the gauge factor converged stably without any significant further increase. The maximum gauge factor was calculated as 104.13 with a standard deviation of 0.06. Five repeated tests on 20 gauges from the same batch yielded an uncertainty of 0.012, confirming the reproducibility and reliability of the sensor.

Figure 5d shows the current–voltage characteristics when a compressive strain was applied to the strain gauge. As strain increases, the slope of the I–V curve (conductivity) initially increases gradually before decreasing, reaching a maximum gauge factor at approximately 0.32% strain, and then converges.

## 4. Conclusions

The presented flexible strain-gauge pressure sensor simultaneously achieves high sensitivity and high linearity by integrating Cr/Au electrodes and a trilayer MoS_2_ thin film onto a PET substrate through a low-temperature process (below 80 °C). The fabricated electrodes maintained a sheet resistance change ratio ΔR/R_0_ below 3% even after 100 bending cycles at a curvature radius of 10 mm, confirming excellent mechanical and electrical stability for flexible applications. The dry-transferred MoS_2_ film exhibited an average roughness of 0.38 nm, indicating successful suppression of residual solvents and polymer contamination.

By employing capacitively coupled plasma etching with O_2_/Ar mixed gas, we achieved high-resolution, large-area patterning of trilayer MoS_2_. This approach reduces process costs and facilitates scalable mass production, outperforming conventional high-temperature or wet processes. XPS analysis confirmed that the Mo 3d and S 2p binding energies match the literature values, demonstrating that chemical integrity was maintained after transfer and patterning. Furthermore, by conducting the entire process below the glass transition temperature of PET (Tg ≈ 80 °C), substrate damage was suppressed while maintaining a stable gauge performance.

The four MoS_2_ gauges attached to the PET diaphragm achieved a representative gauge factor of approximately 104 in dual compressive-tensile mode, representing more than a fifty-fold improvement over commercial metal-foil gauges (GF ≈ 2). The sensor configured as a full bridge was sensitive to kilopascal-range pressure variations, with response and recovery times measured within 200 and 180 ms, respectively, underscoring its suitability for real-time pressure monitoring.

These results demonstrate that an integrated approach combining low-temperature processing, plasma patterning, and 2D channel integration can overcome the limitations of conventional metal foil gauges. This platform has the economic and technical potential to accelerate commercialization in various applications, such as vehicle seat occupancy detection, wearable physiological monitoring, and robotic tactile sensors.

## Data Availability

All data generated or analyzed during this study are included in this published article.
